# Impact of the Result of Soccer Matches on the Heart Rate Variability of Women Soccer Players

**DOI:** 10.3390/ijerph18179414

**Published:** 2021-09-06

**Authors:** Rosa Mª. Ayuso-Moreno, Juan Pedro Fuentes-García, Hadi Nobari, Santos Villafaina

**Affiliations:** 1 Faculty of Sport Science, University of Extremadura, Avda. Universidad S/N, 10003 Cáceres, Spain; roayusom@alumnos.unex.es; 2 Sports Scientist, Sepahan Football Club, Isfahan 81887-78473, Iran; hadi.nobari1@gmail.com; 3 HEME Research Group, Faculty of Sport Sciences, University of Extremadura, 10003 Cáceres, Spain; 4 Physical Activity and Quality of Life Research Group (AFYCAV), University of Extremadura, 10003 Cáceres, Spain; svillafaina@unex.es

**Keywords:** female, football, autonomic modulation, fatigue, training load

## Abstract

The present study aimed to evaluate the effects of a match lost and a match won on post-competitive heart rate variability (HRV) in semi-professional female soccer athletes. A total of 13 players, with a mean age of 23.75 (5.32), from the Cáceres Women Football Club of the Spanish Second National Division participated in our study. They were evaluated in two microcycles which correspond to a match lost and a match won. For each microcycle, baseline and post-competitive measures were collected. Results indicate that HRV was significantly reduced before a match lost and won. Significant differences in HRV variables were observed when compared the lost match, and the match won. Results highlight the importance and usefulness of analyzing the HRV as an indicator of post-competitive fatigue in semiprofessional soccer players. Therefore, a competition’s results could be considered a relevant variable to consider when programming training load.

## 1. Introduction

Biological signals are used as tools for controlling and evaluating training loads and acute and chronic effects on the athlete’s body [1,2]. According to the scientific literature and as technology advances, there is growing interest in monitoring training loads in athletes to control their adaptation [3]. In team sports, monitoring training load is crucial for optimizing performance and preventing injuries, enabling us to anticipate the emergence of overtraining [4,5]. Thus, biomarkers that provide us information regarding changes in athlete’s fatigue are highly appreciated.

Heart rate variability (HRV) is a noninvasive index that evaluates the balance between sympathetic and parasympathetic activity due to the study of successive heartbeats variation over an interval of time [6]. A reduced HRV, induced by a predomination of sympathetic activity, has been related to a reduced regulatory capacity to adapt to different challenges such as exercise or cognitive stressors [7]. Furthermore, a decrease in HRV has been considered a marker of fatigue, poor cardiovascular adaptation to effort, and overtraining [8,9], and, consequently, it has also been correlated with low sports performance [10].

Technological advances have made it possible to improve the fatigue management of athletes through the analysis of HRV [3,9]. HRV has been used in team sports as a sport-specific indicator based on data extracted from training and competitions [11]. In this regard, the study of HRV in team sports is crucial for optimizing performance and preventing injuries, fatigue, or overtraining [12]. In soccer, previous studies have used the HRV to control and manage training load during seasons [13,14,15,16,17,18]. However, in female soccer players, studies which analyze HRV are limited. In this regard, previous studies used the HRV to control training load [19], fatigue [20], or precompetitive anxiety [21].

Fatigue induced by sport is a usual situation within training and competition, but if it is not controlled, it can lead to negative alterations [22]. It is a process that has an effect on some variables of physical performance (technique or precision), and that must be taken into account in football training and recovery [23,24,25]. In this regard, a previous study showed that depending on the quality of the teams against which one competes, the distance and the intensity are different, which influences the players’ fatigue [26]. Furthermore, HRV is also sensitive to cognitive processes [27,28], such as emotions [29]. In this regard, previous studies have reported that athletes experienced mood and wellbeing changes after a loss [30,31]. Specifically, athletes reported higher depression, anxiety, social dysfunction, and anger after a loss, compared to a win, while lower levels of vigor were observed after a loss, compared to after a win [31]. Furthermore, previous studies have found that soccer players who lost a match significantly performed higher distance sprinting and high-speed running than those players who won the match [32]. These findings, together, could suggest that the results of a competition might have a significant impact on the players’ HRV.

Nevertheless, to the best of our knowledge, no previous study has investigated the impact of the results of a soccer competition on the HRV of female soccer players. Given the preceding, the purpose of this study was to investigate if the results of competition could impact the HRV of female soccer players. Thus, a follow-up of soccer players during two weeks of the league, using an HRV recorded two days before the competition (baseline) and one recorded after the competition (post-competition), was conducted. We hypothesized that HRV would be decreased after both matches (a lost and a won match). Nevertheless, significant differences are found in a lost soccer match when compared with a won match.

## 2. Materials and Methods

### 2.1. Participants

A total of 14 players were assessed for eligibility. However, 13 female players (age = 23.76 (5.32), *n* = 13) from the Spanish Second National Division soccer league team participated in this cross-sectional study (see Table 1). One participant could not complete the procedures since she was injured, so she was excluded from the statistical analyses. None of the participants manifested sickness in the match’s week, and no intercurrence was registered.

Participants who complete the procedures had three sessions per week of 1 h and a half, and they had been participating in football competitions an average of 8.85 (2.85) years. All participants agreed and gave written consent to participate in the study. In addition, procedures were approved by the University of Extremadura research ethics committee (approval number: 180/2019).

### 2.2. Procedure

Standardized procedures and recommendations for assessing and reporting HRV results were followed [33]. Due to coach requirements, in terms of time limitation, we decided to conduct a short-term record (5 min) for each HRV (baseline and post-competition). Nevertheless, five minutes of HRV is considered as the gold standard for short-term measurements [34], and it has shown excellent reliability for relevant variables such as RMMSD (ICC = 0.97 (0.81–0.99)) [35].

The participants’ HRV was evaluated before (baseline) and after (post-competition) two matches, in the local dressing room with controlled temperature and humidity (22.3 (1.0) °C; 46.4 (2.8)%) at rest in a sitting position. In order to avoid distractions or interactions between players, at the time of HRV assessment, the room was calmed, all the players were at their places, and they were encouraged to remain silent (without talking).

The HRV measurements were on the same day of the week and at the same time for the two matches and training sessions. Players were familiarized with the procedures and environment. All participants underwent the same training, as well as the same precompetitive routines during the two selected microcycles. Moreover, participants did not take any drug, drink, or other substance that could affect the nervous system 24 h before undergoing the protocol.

**Figure 1:**
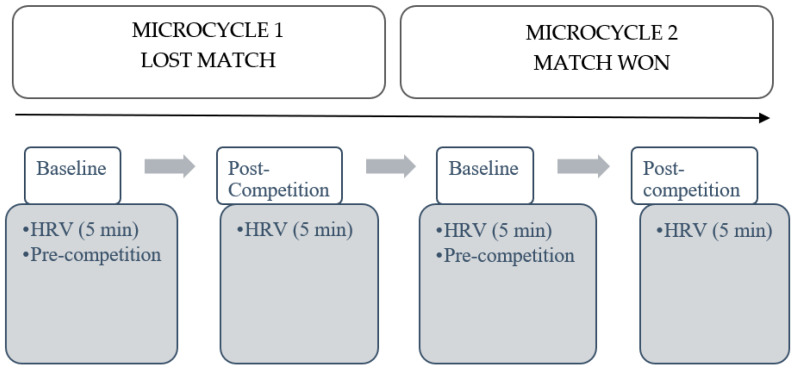
Study procedure timeline. HRV: Heart rate variability.

The same following procedures were carried out in each match (Figure 1):

(1) Baseline: HRV data was recorded two days before the match (in order to avoid pre-competition anxiety response). All participants were evaluated in the same training session before the warm-up to obtain the baseline data of the players. This data was used to normalize the data obtained in the post-competition measure.
(2) Post-competition: HRV data was recorded immediately after the match in a 5-minute register.

One match was lost, and the other was won, so this allowed us to evaluate if the psychophysiological impact is different between a lost or a won match.

### 2.3. Instruments and Outcomes

The HRV data was assessed using the Polar RS800CX (Polar Electro Ltd., Kempele, Finland) heart rate monitor [36] during 5 min at a sampling frequency of 1000 Hz without controlling breathing. HRV data was analyzed using the software Kubios HRV software (v. 3.3; Kubios Oy, Kuopio, Finland) [37]. A middle filter was applied to correct possible artefacts, identifying those R-to-R waves intervals shorter/longer than 0.25 s, compared to the average of the previous beats. Correction replaces the identified artefacts with cubic spline interpolation.

Time, frequency, and nonlinear domains were analyzed: (1) time domain, such as mean heart rate (mean HR), RR intervals, RR50 count divided by the total number of all RR ranges (Pnn50), and the square root of differences between adjacent RR intervals (RMSSD); (2) frequency domain, including low-frequency (LF, 0.04–0.15 Hz) and high-frequency (HF, 0.15–0.4 Hz) ratio (LF/HF) and total power; and (3) nonlinear measures, such as RR variability from heartbeat to short-term Poincaré graph (width) (SD1) and RR variability from heartbeat to long-term Poincaré graph (length) (SD2). Moreover, the stress index, the parasympathetic nervous system index (PNS index), and the sympathetic nervous system index (SNS index) were calculated. The stress index is the square root of the Baevsky’s stress. The PNS index was calculated based on mean RR (ms), RMSSD (ms), and SD1 (%). The SNS index was calculated based on mean HR (bpm), Baevsky’s stress index, and SD2 (%).

### 2.4. Statistical Analysis

The IBM SPSS (Statistical Package for Social Sciences, version 25; IBM, Armonk, NY, USA) statistical package was used to analyze the data.

The Shapiro–Wilk test was conducted to explore the distribution of the HRV data since the sample size was under 50 [38]. Taking into account both the results of this test (see Supplementary Table S1) and the small sample size, nonparametric analyses were conducted. Thus, Wilcoxon signed-rank tests were conducted to explore differences between baseline and post-competitive HRV measures in both the loss and the win matches.

Subsequently, HRV data was normalized for each match (calculating the difference between match and baseline data for each HRV variable). Once HRV variables were normalized, Wilcoxon signed-rank tests were performed to explore the differences between the loss and the win matches. The effect sizes (r) were calculated. It is classified as follows: 0.5 is a large effect, 0.3 is a medium effect, and 0.1 is a small effect [39,40].

## 3. Results

Table 2 shows the values of the HRV at baseline and after losing a soccer match. Significant differences between baseline and post-competition HRV values were found. In this regard, HRV significantly decreased all the studied variables (*p*-value < 0.05).

Table 3 shows the comparison between baseline and post-competition HRV values after winning a soccer match. Significant differences were found in PNS index, SNS index, stress index, mean HR, RR, Pnn50, RMSSD, and SD1 (*p*-value < 0.05).

Comparisons between normalized HRV data in both the loss and the win matches are shown in Table 4. After losing a match, participants showed significant decrease in the PNS index, Pnn50, RMSSD, HF, total power, SD1, and SD2, and an increased SNS index and stress index (*p*-value < 0.05).

## 4. Discussion

The present article aimed to assess the effects of a lost match and a won match on the post-competitive HRV in female soccer athletes. We hypothesized that HRV would be decreased after a lost soccer match compared to a won match. Results showed that HRV significantly decreased after both a lost and a won match. In this regard, comparing the impact of the two matches (a won and a lost match), a lost match induced a significant decrease in HRV variables (RR, pNN50, RMSSD, total power, SD1, and SD2) compared with the post-competitive HRV values obtained after the match won.

Among the tools studied to assess fatigue, HRV has emerged as a helpful tool that provides an indirect evaluation of the balance between sympathetic and parasympathetic nervous systems [34]. Thus, HRV monitoring has been used to prevent overtraining or to manage fatigue in sports such as soccer [13,14] or basketball [41]. Our results showed that a lost match induced a decrease in HRV variables such as RR, pNN50, RMSSD, total power, SD1, and SD2. In this regard, previous studies have highlighted the importance of RMSSD in the identification of fatigue [9,18]. In the same line, Proietti, di Fronso, Pereira, Bortoli, Robazza, Nakamura and Bertollo [18] showed that RMSSD is a useful HRV variable to control the training effects in professional soccer players. Therefore, these results suggest that this variable would be quite interesting in managing fatigue in female soccer players.

The physical and mental impact of losing a soccer match could explain the results obtained. Regarding the impact of physical load in the HRV, previous studies have found that increasing physical activity intensity can significantly impact the HRV [42]. A reduced HRV after a stressor itself (a soccer match) had ceased can be due to homeostatic processes. In this regard, gluconeogenesis would presumably take place, which allows muscles and the liver to refill their energy substrates. This state would require an extra cardiac output, so a higher HR, mediated by an increase in the sympathetic modulation [43,44], can be expected. In addition, the sympathetic modulation can be significantly impacted by proinflammatory cytokines [44] induced by exercises of high duration and intensity [45]. In this line, a previous study showed that soccer players who lose a match significantly performed higher distance sprinting and high-speed running than those who won the match [32]. This could explain that players showed reduced HRV after losing a match.

Regarding the impact of mental processes on HRV, previous studies have reported that athletes experienced mood and wellbeing changes after a match loss [30,31]. Athletes reported higher depression, anxiety, social dysfunction, and anger after a loss than a win, while lower levels of vigor were observed after a loss, compared to after a win [31]. Taking into account the impact of anxiety [46] or depression [47] on the HRV, as well as the role of emotions on HRV [29], decreases in HRV variables could also be justified by this reason. Therefore, the results of a competition could be considered a relevant variable to consider when programming training load. However, HRV can be considered as an index of overall fatigue, where physical and mental states can be affected. Thus, our results should be taken with caution, since the evaluation only assessed before and after two matches, and the total volume of completed load was not taken into account. Therefore, it would be necessary to independently evaluate the total volume of completed load due to the significant impact of physical load on the HRV [48]. Future studies should independently explore the role of mental and physical components in reducing HRV after a lost match.

Results indicate that five-minute pre-and post-game HRV measurements appear to be a useful way of monitoring the state of sympathetic and parasympathetic balance in female soccer players. This is relevant, since the analysis of this monitoring would be helpful to prevent fatigue by managing training loads before matches. In this regard, previous studies showed that variables such as RMSSD or the stress index are biomarkers of internal load, and therefore are sensitive enough to detect fatigue [49,50]. This is extremely useful since the player cannot voluntarily alter HRV results, unlike subjective scales such as wellness or stress questionnaires [51]. However, different protocols of HRV monitoring during sports seasons can be found in the scientific literature [13,14,15,16,17,18]. In this regard, Ravé and Fortrat [13] conducted the HRV analysis while players performed a 10 min phase in the supine position followed by a 7 min standing phase during a 5-week training period. Thorpe, Strudwick, Buchheit, Atkinson, Drust and Gregson [15] recorded the HRV during 5 min seated after 5 min cycling/5 min recovery at 130 W (85 rpm). Boullosa, Abreu, Nakamura, Muñoz, Domínguez and Leicht [14] assessed the weekly HRV, from the mean of four-daily, continuous 3 h nighttime recordings. However, there are similar protocols, but not identical to ours (5 min at rest). In this regard, Vilamitjana, Lentini, Pérez-Júnior and Verde [16] measured the HRV during 5 min immediately after awakening on the match day. Botek, Krejčí, McKune and Klimešová [17] recorded 300 artefact-free subsequent RR intervals at rest. In the same line, Proietti, di Fronso, Pereira, Bortoli, Robazza, Nakamura and Bertollo [18] recorded 10 min seated. However, any of the protocols can be measured before and after the match. Taking into account the variability in the procedures, future studies should explore the feasibility of these protocols, trying to standardize them. This would allow the comparison with other studies.

One limitation of the present study is the relatively small sample size. Nevertheless, soccer is a sport discipline of great relevance and impact on society, and women are increasing their presence in this game. However, interfering with the dynamics of individual players on a training or match day is a drawback when it comes to obtaining a larger number of women participants. We were aware that it would be difficult for volunteers to complete the study due to, for example, injuries (a player was lost for this reason) or poor individual play. For all these reasons, we considered that, given the magnitude of the championship (second division of the Spanish female soccer league), the sample to be studied and the results obtained are of enormous interest. Furthermore, the evaluation of HRV only in relation to the results of the competition (win or lose) is very partial, and future studies should also evaluate the total volume of completed load, which affects the spectrum HRV. Moreover, it would be interesting to analyze the impact that the player’s position on the field (i.e., goalkeeper, central, midfield, or forward) can have on the HRV [52,53,54,55]. It is possible that a different impact on HRV can be observed depending on the aerobic and anaerobic requirements for each position on the field. Therefore, continuous and systematic analyses throughout the season can allow for individual monitoring of each player, providing valuable information for adjusting training loads and/or detecting possible interventions on a psychological level [11,21,56]. Lastly, due to time limitation and staff´s requirement, HRV assessment lasted 5 min. Although it is the gold standard for short-term measurements [34], future studies should investigate the specific reliability of this assessment in female soccer players.

## 5. Conclusions

The result of a soccer competition might significantly impact the HRV of female soccer players. In this regard, a lost match led to a decrease in HRV when compared with a match won. Therefore, researchers, coaches, or physical trainers should take into account the results of a competition when programming training load, since fatigue might be higher after a lost soccer match.

## Figures and Tables

**Table 1:** Descriptive data of participants.

Variable	Mean (SD)
Age (years)	23.75 (5.32)
Competition experience (years)	8.85 (2.85)
Height (cm)	163.61 (5.54)
Weight (kg)	58.30 (7.54)
Body Mass Index (kg/m^2^)	21.78 (2.63)

**Table 2:** HRV at baseline and after losing a soccer match for female soccer athletes.

Variable	Baseline Mean (SD)	Post-Competition Mean (SD)	*p*-Value	Effect Size
PNS Index	0.40 (1.69)	−2.42 (0.48)	0.002 *	0.843
SNS Index	0.55 (2.98)	4.45 (1.84)	0.013 *	0.688
Stress Index	9.63 (8.31)	19.93 (8.40)	0.016 *	0.669
Mean HR	72.95 (20.06)	104.93 (10.94)	0.002 *	0.862
RR	860.26 (154.03)	578.06 (65.35)	0.002 *	0.862
pNN50	41.51 (20.72)	3.32 (4.62)	0.002 *	0.843
RMSSD	66.84 (34.23)	16.72 (9.51)	0.002 *	0.843
HF	50.17 (16.76)	13.06 (9.29)	0.002 *	0.843
LF	49.74 (16.80)	86.90 (9.32)	0.002 *	0.843
LF/HF	1.36 (1.37)	11.43 (9.24)	0.002 *	0.843
Total power	4157.06 (3956.21)	682.13 (711.64)	0.002 *	0.862
SD1	47.34 (24.25)	11.83 (6.73)	0.002 *	0.843
SD2	73.07 (33.71)	34.45 (16.47)	0.002 *	0.862

* *p*-value < 0.05; HR: heart rate; RR: time between intervals R–R; pNN50: percentage of intervals > 50 ms different from the previous interval; RMSSD: the square root of the mean of the squares of the successive differences of the interval RR; LF/HF: low-frequency (LF) (ms2)/high-frequency (HF) (ms2) ratio; total power: the sum of all the spectra; PNS index: parasympathetic nervous system index, SNS index: sympathetic nervous system index and stress index; SD1: dispersion, standard deviation, of points perpendicular to the axis of line-of-identity in the Poincaré plot; SD2: dispersion, standard deviation, of points along the axis of line-of-identity in the Poincaré plot.

**Table 3:** HRV at baseline and after winning a soccer match for female soccer athletes.

Variable	Baseline Mean (SD)	Post-Competition Mean (SD)	*p*-Value	Effect Size
PNS Index	−0.64 (1.30)	−1.83 (0.97)	0.019 *	0.652
SNS Index	1.11 (1.60)	2.79 (1.90)	0.015 *	0.674
Stress Index	10.73 (4.23)	14.49 (6.03)	0.041 *	0.565
Mean HR	79.52 (14.63)	94.10 (14.59)	0.015 *	0.566
RR	776.95 (134.80)	653.20 (110.65)	0.023 *	0.631
pNN50	21.36 (20.10)	7.67 (10.73)	0.023 *	0.630
RMSSD	42.97 (24.91)	24.21 (15.53)	0.028 *	0.609
HF	37.45 (19.84)	16.27 (7.60)	0.006 *	0.762
LF	62.38 (19.87)	83.71 (7.58)	0.006 *	0.762
LF/HF	2.91 (3.41)	6.65 (4.02)	0.028 *	0.609
Total power	1956.09 (1698.56)	1668.89 (1608.24)	0.272	0.304
SD1	30.43 (17.65)	17.14 (11.00)	0.028 *	0.609
SD2	56.28 (23.42)	49.25 (22.56)	0.182	0.370

* *p*-value<0.005; HR: heart rate; RR: time between intervals R–R; pNN50: percentage of intervals > 50 ms different from the previous interval; RMSSD: the square root of the mean of the squares of the successive differences of the interval RR; LF/HF: low-frequency (LF) (ms2)/high-frequency (HF) (ms2) ratio; total power: the sum of all the spectra; PNS index: parasympathetic nervous system index, SNS index: sympathetic nervous system index and stress index; SD1: dispersion, standard deviation, of points perpendicular to the axis of line-of-identity in the Poincaré plot; SD2: dispersion, standard deviation, of points along the axis of line-of-identity in the Poincaré plot.

**Table 4:** Impact on heart rate variability of a loss and a win soccer match.

Variable	Loss Match	Win Match	*p*-Value	Effect Size
Heart Rate Variability				
PNS Index	−2.83 (1.64)	−1.06 (1.31)	0.019 *	0.549
SNS Index	3.89 (3.20)	1.47 (1.82)	0.028 *	0.610
Stress Index	10.30 (11.59)	2.64 (6.15)	0.019 *	0.649
mean HR	31.97 (17.60)	7.34 (27.14)	0.023 *	0.630
RR	−282.19 (137.09)	−173.99 (2625.27)	0.116	0.436
pNN50	−38.18 (22.40)	−14.29 (20.90)	0.023 *	0.630
RMSSD	−50.12 (34.73)	−20.62 (26.07)	0.033 *	0.591
HF	−37.11 (22.28)	−22.43 (22.31)	0.152	0.397
LF	37.16 (22.32)	14.88 (25.55)	0.055	0.533
LF/HF	10.07 (9.78)	3.22 (5.20)	0.055	0.533
Total power	−3474.93 (3524.27)	−415.57 (1706.29)	0.001 *	0.882
SD1	−35.50 (24.60)	−14.60 (18.47)	0.033 *	0.591
SD2	−38.62 (24.88)	−10.81 (27.12)	0.016 *	0.669

* *p*-value < 0.05; HR: heart rate; RR: time between intervals R–R; pNN50: percentage of intervals > 50 ms different from the previous interval; RMSSD: the square root of the mean of the squares of the successive differences of the interval RR; LF/HF: low-frequency (LF) (ms2)/high-frequency (HF) (ms2) ratio; total power: the sum of all the spectra; PNS index: parasympathetic nervous system index, SNS index: sympathetic nervous system index and stress index; SD1: dispersion, standard deviation, of points perpendicular to the axis of line-of-identity in the Poincaré plot; SD2: dispersion, standard deviation, of points along the axis of line-of-identity in the Poincaré plot.

## Data Availability

Data will be available upon reasonable request to the corresponding author.

## Supplementary Materials

The following are available online at https://www.mdpi.com/article/10.3390/ijerph18179414/s1, Table S1: Shapiro-Wilk test.
