# Supplementary material for: Impact of the Result of Soccer Matches on the Heart Rate Variability of Women Soccer Players
Source: Int J Environ Res Public Health. 2021 Sep 6;18(17):9414. doi: 10.3390/ijerph18179414 (PMC8430763; doi:10.3390/ijerph18179414)
Supplement: Supplementary file 1 [file ijerph-18-09414-s001.zip › ijerph-1365171-supplementary.pdf]

Supplementary Table S1. Shapiro-Wilk test

Variable	Baseline Win match (p-value)	Baseline Loss Match (p-value)	After win match (p-value)	After lose match (p-value)
PNS Index	0.046	0.023	0.005	0.039
SNS Index	0.003	0.046	0.125	0.015
Stress Index	0.012	0.056	0.068	0.049
mean HR	0.057	0.045	0.062	0.050
RR	0.005	0.069	0.120	0.046
pNN50	0.025	0.040	0.096	0.026
RMSSD	0.042	0.036	0.120	0.051
HF	0.080	0.045	0.074	0.035
LF	0.095	0.020	0.056	0.046
LF/HF	0.075	0.038	0.064	0.023
Total power	0.045	0.041	0.051	0.036
SD1	0.005	0.001	0.025	0.021
SD2	0.008	0.009	0.042	0.015
